# Is there an association among spirituality, resilience and empathy in medical students?

**DOI:** 10.1186/s12909-024-05687-6

**Published:** 2024-06-28

**Authors:** Anna TMS Moura, Andreia M. Coriolano, Renata Kobayasi, Silvio Pessanha, Hellen LMC Cruz, Suely M. Melo, Inah MD Pecly, Patricia Tempski, Milton A. Martins

**Affiliations:** 1 Faculdade de Ciências Médicas, Universidade Estadual do Rio de Janeiro, Rio de Janeiro, Brazil; 2 Centro de Desenvolvimento de Educação Médica, Faculdade de Medicina, Universidade de São Paulo, Avenida Dr. Arnaldo 455 sala 1210, Sao Paulo, Brazil; 3 Instituto de Educação Médica, IDOMED, Rio de Janeiro, Brazil

**Keywords:** Medical education, Undergraduate, Spirituality, Resilience, Empathy

## Abstract

**Background:**

Spirituality has religious and nonreligious dimensions and is often linked to well-being, positive emotions, connection and meaning in life. Both empathy and resilience are important in medical training and future professional practice since they are considered core skills related to professionalism and patient care. Our study aimed to understand the relationships among spirituality, resilience, and empathy in medical students. We also aimed to determine whether there are differences by gender and between medical students in different years of a medical program.

**Methods:**

Medical students (*n* = 1370) of the first to fourth years of a six-year medical program, from six medical schools, completed questionnaires to assess empathy (Jefferson Empathy Scale and Davis Multidimensional Interpersonal Reactivity Scale) and resilience (Wagnild & Young Scale) and to rate their spirituality.

**Results:**

Medical students with high spirituality showed higher scores for both resilience and empathy (*p* < 0.001). In addition, we observed higher levels of both spirituality and empathy, but not resilience, in female medical students than in male medical students. In contrast, we did not detect significant differences in spirituality, empathy, or resilience between students in different years of medical school.

**Conclusion:**

Medical students with high levels of spirituality have also higher scores for both empathy and resilience. Spirituality, empathy and resilience have similar values for students in different years of a medical program.

## Background

Spirituality has religious and nonreligious dimensions and is often linked to well-being, positive emotions, connection and meaning in life [1, 2]. The concept of spirituality is broad and involves connections with others, altruism, and a search for meaning and purpose in life. In medical practice, it is related to better health care outcomes due to respect for the religious and spiritual needs of patients [1–4]. Spirituality is a complex concept that varies considerably depending on cultural, religious, and academic influences. In recent decades, spirituality has become increasingly important in health care and health professional education, since it has been found to be related to less substance abuse, lower incidence of depression, better ability to cope with disease and treatment adherence, and lower mortality rates [5–7]. Previous studies have shown an association between openness to spirituality, higher levels of empathy and lower anxiety in medical students [8].

Resilience is the dynamic capacity to positively face and overcome adversity through personal transformation and growth [9]. Resilience has been defined as a person’s capacity to resist adversity without developing physical, psychological, or social disabilities [9]. Resilience is an emotional competence and can be considered a behavior that can be acquired and improved. It has been suggested that resilience involves cognitive processes that encompass at least four dimensions: self-efficacy, planning, self-control, and commitment and perseverance [10]. Medical students with higher levels of resilience have a better perception of their quality of life and educational environment [11]. It is important to understand what can facilitate the development of resilience, that is required of medical students in training when facing stressful and emotionally demanding situations [11, 12].

In the same way, it is important to understand how to teach and assess empathy among medical students during medical courses. Empathy is a complex construct that has cognitive and affective components. Hojat et al. defined empathy in patient care situations as “a cognitive attribute that involves an ability to understand the patient’s inner experiences and perspective and a capability to communicate this understanding” [13]. Mehrabian et al. defined the affective component of empathy as “an individual’s vicarious emotional response to perceived emotional experiences of others.” [14] Empathy is one of the primary competencies in health professionals’ training due to its relevance in professional-patient relationships and can influence patient satisfaction and confidence in proposed treatment [15–17].

Both empathy and resilience are important in medical training and future professional practice and are considered core skills related to professionalism and patient care [3, 9, 18]. We reasoned that medical students with high spirituality have higher levels of resilience and empathy. In the present study we evaluated the relationships among medical students’ spirituality, resilience, and empathy. We adopted the definition of spirituality by Koenig et al. [1, 19] “Spirituality is the personal quest for understanding answers to ultimate questions about life, about meaning, and about relationship to the sacred or transcendent, which may (or may not) lead to or arise from the development of religious rituals and the formation of community”. We also aimed to understand whether there was a difference in resilience, empathy and spirituality based on gender and different years of medical education.

## Methods

### Study design and setting

The study is part of a cross-sectional, multicenter study that involved six medical schools from the same educational organization (Instituto de Educação Médica – IDOMED) in different regions of Brazil. Data were collected from February to May of 2023 from six medical schools in the cities of Rio de Janeiro, Juazeiro do Norte, Jaraguá do Sul, Angra dos Reis, Alagoinhas and Teresina.

The study was developed after approval by the Research Ethics Committee of the institution (CAAE: 58284522.0.0000.5259). All participants signed the informed consent form, which explained the risks and benefits and guaranteed confidentiality and the right to refuse and withdraw their participation. After the informed consent form was signed, it was automatically sent to the email registered by the participant. Data were collected with a form on the Google Forms platform.

The questionnaires were administered during class time. The local research coordinators were responsible for using an infographic to publicize the study among the academic community, including teachers from different areas and class leaders from each period of the first four years of medical course. All students who were regularly enrolled in medical courses and were present in the classroom were invited to participate.

### Instruments

#### Sociodemographic questionnaire

The survey was organized to identify students’ demographic characteristics, such as age, gender, and the year of their medical course.

#### Empathy measurement

Two instruments were used to assess empathy: the Jefferson Empathy Scale (JES) and the Davis Multidimensional Interpersonal Reactivity Scale (EMRI) [20, 21]. The JES assesses empathy in doctor‒patient relationships. It presents twenty questions with answers on a Likert scale ranging from one to seven. The EMRI assesses the affective, cognitive, and behavioral components of empathy and includes three scales for these subdomains: empathic consideration, perspective taking and personal distress. It uses twenty-one questions with answers on a Likert scale from one to five. Higher scores on these instruments indicate greater empathic tendencies. Both instruments have been previously translated to and validated for Brazilian Portuguese [22, 23]. JES had a Cronbach’s alpha of 0.854 and Davis Scale had a Cronbach’s alpha of 0.806.

#### Resilience measurement

The Wagnild and Young scale (abbreviated version) was used [24]. This scale has 14 statements on a seven-point Likert scale. Higher scores suggest greater resilience. Although this questionnaire has various domains, in this study, we used only the global score. This scale has been previously translated to and validated for Brazilian Portuguese [25]. This questionnaire had a Cronbach’s alpha of 0.934.

#### Self-evaluation of spirituality

We included the definition of spirituality of Koening et al. [1, 19] in the questionnaire and students were asked if, according to this definition, they considered themselves to have (a) no spirituality, (b) low spirituality, (c) moderate spirituality or (d) high spirituality. The results were analyzed using three groups: (a) no or low spirituality, (b) moderate spirituality and (c) high spirituality. We combined the groups of students that answered to have no spirituality and low spirituality because in the first analysis we did not observe any difference in their scores of resilience and empathy. Students who responded “I don’t know” to the spirituality question were excluded from the analysis.

### Statistical analysis

Descriptive statistical analysis was used for students’ sociodemographic categorization (frequency), scores on the empathy and resilience questionnaires (mean, standard deviation) and frequency distribution related to gender and the year of the medical program.

Student’s t-test was used to compare values of two groups and ANOVA followed by Bonferroni analysis was used to compared three groups. The chi-square test was used to compare the distribution of frequencies. When we used chi-square to compare frequencies among three groups (spirituality groups), we performed a correction for multiple comparisons. We established the level of statistical significance as 0.05. All statistical analyses were performed using SPSS Statistics for Windows, version 22.0 (released 2013, IBM Corp, Armonk, NY).

## Results

The final analysis included 1,370 valid responses, that were 54.7% of the total number of medical students of the 1st to 4th year of medical course of the medical schools studied (2,504 students). Among the students that were present in the classroom when the researchers made the invitation to participate in the study, only six medical students refused to participate.

The average age of the respondents was 25.3 ± 7.0 years and ranged between 17 and 57 years. Women accounted for 63.6% of the total. In relation to the year of the course, 54.1% of the respondents were in the first two years and 45.9% were in the third and fourth years.

Table 1 shows the results of the empathy scales (total values and comparisons between genders). Women showed significantly greater empathetic tendencies than men for all three domains of the Davis Empathy Scale and the total score of the Jefferson Scale (*P* < 0.001 for all comparisons). For the gender comparison, we included 1,366 students because four students did not respond to the question about gender.

Table 1 shows the total scores of the resilience questionnaire and percentages of the distribution at different levels, identified as very low or low (14 to 64 points), moderately low or moderately high (65 to 81 points) and high or very high (82 to 98 points) resilience. We did not observe significant differences when comparing the resilience values or percentages between female and male medical students.

Table 1 also displays the percentages of students who considered themselves to have no or low spirituality, moderate spirituality, or high spirituality. Those who answered “I do not know” to the question concerning spirituality were excluded (37 students). We observed that the percentage of female medical students who responded “high” to the question about spirituality was significantly greater than that of male medical students (36.3% and 25.6%, respectively, *P* < 0.001).

Table 2 shows the comparisons between medical students who were in their first or second years and those in their third or fourth years. Only one student did not report his or her year of medical school. We did not observe a statistically significant difference in the results of the questionnaires, including the empathy scale, the resilience scale and different levels of spirituality.

We also compared the results of the empathy and resilience scales considering different levels of spirituality (Table 3). We observed significant differences with regard to both empathy and resilience, with medical students with high spirituality also showing higher values for resilience and empathy. Medical students with higher levels of spirituality had significantly higher scores for the two domains of the Davis Scale (empathic concern and perspective taking) and total scores on the Jefferson Scale than those with moderate or no/low spirituality (*P* < 0.001 for all comparisons). The only domain of the Davis Scale that did not show a statistically significant difference among groups in terms of the level of spirituality was personal distress.

We also observed significantly higher scores on the resilience scale for medical students with high spirituality (72.5 ± 18.3, 76.0 ± 17.6 and 78.8 ± 18.2, mean values of resilience, respectively, for students with no/low, moderate and high spirituality, respectively; *P* < 0.001). The percentage of medical students with high or very high resilience scores was also significantly greater in the high spirituality group (36.4%, 46.9% and 56.9%, respectively, for students with no/low, moderate and high spirituality, *P* < 0.001).

To better understand the relationship between higher resilience values and high spirituality, we compared the percentage of “totally agree + agree” responses on each of the fourteen items of the resilience questionnaire between students with no or low spirituality and those with high spirituality (Table 4). A greater percentage of students with high spirituality responded “totally agree and agree” to eleven items. Only three items did not show a statistically significant difference in positive responses (“I usually manage one way or another”, “I feel that I can handle many things at a time”, and “I can usually find something to laugh about”).

**Table 1:** Spirituality, empathy, and resilience in Brazilian medical students

	Total	Men	Women	*P* value
**Number of medical students**	1366	497 (36.4%)	869 (63.6%)	
Davis empathy scale *				
Empathic concern	67.9 (16.7)	61.4 (16.2)	71.6 (15.8)	< 0.001
Perspective taking	64.8 (17.2)	63.2 (16.8)	65.7 (17.3)	< 0.001
Personal distress	40.5 (17.3)	36.2 (16.0)	42.9 (17.5)	< 0.001
Jefferson empathy scale*	114.7 (16.0)	111.0 (16.8)	116.9 (15.0)	< 0.001
Resilience scale*	75.8 (18.3)	75.3 (19.2)	76.2 (17.7)	NS
Very low/low#	280 (20.5%)	107 (38.2%)	173 (61.8%)	NS
Moderately low/high#	440 (32.2%)	154 (35.0%)	286 (65.0%)	
High/very high#	646 (47.3%)	236 (36.5%)	410 (63.5%)	
**Spirituality**#				
No or low	318 (23.9%)	159 (33.4%)	159 (18.6%)	< 0.001
Moderate	580 (43.6%)	195 (41.0%)	385 (45.1%)	
High	432 (32.5%)	122 (25.6%)	310 (36.3%)	

* Scores are means and standard deviations; # percentages. Means were compared by Student’s t-test. Percentages were compared using chi-square test.

**Table 2:** Spirituality, empathy and resilience in Brazilian medical students, comparing first/second years with third/fourth years

	First and second years	Third and fourth Years	*P* value
**Number of medical students**	741	628	
Davis empathy scale *			
Empathic concern	68.0 (16.6)	67.6 (16.8)	NS
Perspective taking	65.1 (17.1)	64.9 (17.3)	NS
Personal distress	39.8 (17.4)	41.4 (17.1)	NS
Jefferson empathy scale*	113.9 (16.2)	115.6 (15.8)	NS
Resilience scale*	75.0 (18.7)	76.7 (17.7)	NS
Very low/low#	163 (22.0%)	119 (18.9%)	NS
Moderately low/high#	239 (32.8%)	203 (32.3%)	
High/very high#	339 (45.7%)	306 (48.7%)	
**Spirituality**			
No or low	181 (25.1%)	140 (22.9%)	NS
Moderate	326 (45.2%)	254 (41.6%)	
High	214 (29.7%)	217 (35.5%)	

* Scores are means and standard deviations; # percentages. Means were compared by Student’s t-test. Percentages were compared using chi-square test.

**Table 3:** Spirituality levels, resilience, and empathy in Brazilian medical students

	Spirituality levels			
	No or low	Moderate	High	*P* value
**Number of medical students**	321	580	432	
Davis empathy scale *				
Empathic concern	63.6 (17.2)	68.3 (15.8)	71.1 (16.9)	< 0.001
Perspective taking	61.9 (17.2)	64.6 (16.9)	67.5 (17.3)	< 0.001
Personal distress	39.6 (16.9)	41.0 (17.6)	40.0 (17.0)	NS
Jefferson empathy scale*	112.2 (16.6)	114.8 (16.0)	117.1 (15.2)	< 0.001
Resilience scale*	72.5 (18.3)	76.0 (17.6)	78.8 (18.2)	< 0.001
Very low/low#	82 (25.5%)	119 (20.5%)	68 (15.7%)	< 0.001
Moderately low/high#	122 (38.0%)	189 (32.6%)	118 (27.3%)	
High/very high#	117 (36.4)	272 (46.9%)	246 (56.9%)	

* Scores are means and standard deviations; # percentages. Means were compared by ANOVA followed by Bonferroni analysis. Percentages were compared using chi-square test.

**Table 4:** Comparison between Brazilian students with no/low spirituality and those with high spirituality, considering each item of the Resilience Scale questionnaire

Item of Resilience Scale	No or low spirituality	High spirituality	Ratio	*P* value
I usually take things in stride.	37.1%	50.5%	1.36	< 0.001
When I’m in a difficult situation, I can usually find my way out of it.	53.9%	71.1%	1.31	< 0.001
My belief in myself gets me through hard times.	45.2%	59.0%	1.30	0.002
I stay interested in things.	43.6%	56.9%	1.30	0.008
My life has meaning.	62.3%	81.0%	1.3	< 0.001
I can get through difficult times because I’ve experienced difficulty before.	55.5%	69.7%	1.25	0.001
I am friends with myself.	51.7%	64.8%	1.25	0.007
In an emergency, I’m someone people can generally rely on.	63.2%	78.2%	1.23	< 0.001
I have self-discipline.	45.2%	54.4%	1.20	0.004
I am determined.	62.6%	74.3%	1.18	0.017
I feel proud that I have accomplished things in life.	66.0%	75.9%	1.15	0.008
I usually manage one way or another.	50.5%	57.9%	1.14	NS
I feel that I can handle many things at a time.	38.9%	43.5%	1.11	NS
I can usually find something to laugh about.	62.6%	68.8%	1.09	NS

We show the percentages of “totally agree” and “agree” responses

## Discussion

This study aimed to evaluate the relationships among spirituality, empathy, and resilience in medical students. We observed that medical students with high spirituality had higher scores for both resilience and empathy. In addition, we observed higher levels of both spirituality and empathy, but not resilience, in female medical students than in male medical students. In contrast, we did not detect significant differences in spirituality, empathy, or resilience among students in different years of medical school.

Medical school is a six-year medical program in Brazil. The first four years are basic and clinical studies, and the last two years are clerkships (“internship”). Medical schools that were studied have a curriculum with integration between basic and clinical studies and social sciences from the first to fourth year of medical course. Medical students have an early contact with patients and the community since the first semester, under the supervision of preceptors and faculty. The last two years of medical school are called “internship”, with rotations where medical students assume direct care of patients, under the supervision of the medical staff. In this study, we evaluated only students in the first four years of the medical course. We decided to study medical students before the “internship” because we aimed to evaluate them during the basic/clinical years, before they assumed a direct care of patients. Our sample was composed of 63.3% female students, reflecting the current profile of Brazilian medical schools.

Students can start medical school just after the equivalent of high school in Brazil. However, there is an increasing number of people that decide to go to medical school later in life. In our study, 341 medical students (24.9% of total) were older than 30 years. We compared the values of resilience, empathy and spirituality of these medical students to the values of younger students, and we did not find any significant difference (data not shown).

Although there is a relationship between religiosity and spirituality, we decided to adopt the nonreligious definition of Koenig et al. when asking medical students to indicate their different levels of spirituality [1, 19]. We did not observe any significant differences in the level of spirituality among students in different years of medical school (Table 2). However, more female medical students considered themselves highly spiritual (36.3 women vs. 25.6% men, *P* < 0.001).

Previous studies have shown that high levels of spirituality are related to a better prognosis, greater adherence to treatment, easier decision-making, and more ethical values [1, 7, 19]. Supporting the understanding of other people’s suffering also favors clinical care by bringing compassion to relationships with patients in all types of scenarios [26]. During medical school, spirituality may be protective against burnout and psychological suffering. Wachholtz and Rogoff [27] observed that students with higher levels of spiritual well-being and daily spiritual experiences described themselves as more satisfied with their life in general, while students with low scores had higher levels of psychological distress and burnout. Lucchetti et al. [28] concluded that many medical students believe that spirituality has an influence on patients’ health and want to address this in clinical practice. Nevertheless, the majority feel they are unprepared to do so, and that medical school does not provide the necessary training. These results suggest that there is a gap between students’ attitudes/needs in this area and the training they are receiving. We suggest that spirituality must be an important content of medical courses, preparing medical students to consider the important impact of spirituality of their patients in health and response to health care.

Resilience refers to the ability to deal with life events, view problems as opportunities for personal growth, and recognize limitations as well as personal and collective resources. It also means being able to organize strategies through self-reflection, creativity, optimism and humor and being flexible and able to act with responsibility and ethical awareness [9, 10]. In recent years, resilience, from the psychological perspective of Howe et al. [12], has been assimilated by health sciences and is associated with better outcomes in health promotion, well-being, and quality of life [11, 29]. In this context, resilience may be linked to improved academic and professional performance. In a recent study, involving a large number of medical students from twenty-two medical schools, we showed that medical students with higher resilience scores have better quality of life and better perceptions of the academic environment [11].

We reasoned that spirituality may play a role in resilience. This hypothesis prompted our group to study the relationship between spirituality and resilience. Because a high prevalence of anxiety and depression among medical students has been reported as a result of academic pressure, workload, financial hardship, sleep deprivation and other stressors, it is important to identify coping strategies [30]. Spirituality can be considered an important resource throughout training that provides support and coping skills for students who experience situations involving emotional stress and relational conflicts in addition to facilitating a better balance between personal life and work [6]. However, this topic has not been well explored in the curriculum and is present mainly in discussions about ethics, palliative and critical care, end-of-life care and some chronic diseases with poor prognoses [3, 28, 31, 32]. There are already some initiatives to include spiritual history taking in the initial years of courses as a component of communication skills [32, 33].

Empathy is an important component of medical professionalism and has frequently been associated with improvements in health outcomes and quality of care in clinical practice [34–37]. Empathy is the ability to share, understand and respond with care to the experiences of others [5, 36, 37]. Empathy involves cognitive and emotional reactions, such as actively listening to, identifying, and understanding the concerns and emotions of others and conveying this understanding.

Our results did not show significant differences between scores of empathy and course years. A systematic review showed inconclusive results regarding changes in the level of empathy throughout years of study [38], with studies suggesting a decrease in empathy and studies suggesting no change. It has been suggested that the changes in empathy during medical school observed in previous studies depend on the region of the world studied [39]. One of the possible reasons that we did not observe changes in empathy in medical students is that, in the medical schools studied, there is an early contact with patients and the community, under the supervision of preceptors and faculty and the values of medical profession are an important aspect of the curricula since the beginning of medical program.

We decided to use the two most commonly used questionnaires, the Jefferson and Davis Scales, to evaluate empathy in medical students. Davis Scale has questions related to empathy in general and Jefferson Scale has questions related to medical profession. We adopted a framework that considers empathy to be a multidimensional construct that consists of cognitive and affective components [21]. All domains of the Davis Scale showed significantly higher scores for female medical students than for male medical students (Table 1), and empathetic concern and perspective taking were at a higher level for medical students with high levels of spirituality (Table 3). One possible factor that explains the association between spirituality and empathy is that these concepts may overlap since they are associated with connection to others or the practice of helping people. In addition, many questions in the resilience inventory explore beliefs that can be connected to the concept of spirituality. To explore this hypothesis, we compared each item between students with high spirituality and those with low or no spirituality (Table 4). The items with greater differences between the two groups are related to the meaning or purpose of life and are characteristic of the concepts of resilience and spirituality.

The development of educational activities to stimulate reflection about spirituality, empathy and resilience during medical courses is considered pivotal for the development of an ethical professional identity for medical students and physicians in training. In the United States, schools of osteopathic medicine always consider the spiritual aspects of medical care [40]. In medical education, discussing spirituality as connected to people (classmates, teachers and patients) and the world can be a good strategy for stimulating respect for equity, diversity and inclusion in medical schools and in the health system [3, 9, 12, 16, 41–45].

Our study has several strengths. It was multicentric, included medical schools in different regions of the country and included a large group of students who were in their first and clinical years. To ensure completion of the long questionnaire, only students present in the classroom were invited to participate in the study. One limitation of the study is its cross-sectional design and lack of follow-up of the participants involved.

## Conclusions

Medical students with high levels of spirituality had also both higher scores of empathy and resilience. We did not observe differences in empathy scores between students with different years of training. Female students had higher scores for both spirituality and empathy but not for resilience. We suggest that spirituality, resilience and empathy become an important part of medical school curricula.

## Data Availability

The datasets generated during and/or analyzed during the current study are available from the corresponding author on reasonable request.
